# Persistent hypoxemia after an asthma attack

**DOI:** 10.1186/s13089-019-0121-z

**Published:** 2019-03-21

**Authors:** Cristian Deana, Laura Conangla, Luigi Vetrugno, Massimiliano Saltarini, Stefania Buttera, Tiziana Bove, Flavio Bassi, Amato De Monte

**Affiliations:** 1 Anesthesiology and Intensive Care 1, Department of Anesthesia and Intensive Care, Azienda Sanitaria Universitaria Integrata–Udine, P.le S.Maria Della Misericordia n. 15, 33100 Udine, Italy; 2 Primary Care Service Barcelonès Nord i Maresme, Catalan Health Institute, Barcelona, Spain; 3 Anesthesiology and Intensive Care Clinic, Department of Medicine, University of Udine, P.le S. Maria Della Misericordia n.15, 33100 Udine, Italy; 4 Anesthesiology and Intensive Care 2, Department of Anesthesia and Intensive Care, Azienda Sanitaria Universitaria Integrata–Udine, P.le S.Maria Della Misericordia n. 15, 33100 Udine, Italy

**Keywords:** Patent foramen ovale, Microbubbles contrast, Echocardiography, Asthma attack

## Abstract

The presence of an unknown intracardiac shunt due to a patent foramen ovale may be an unusual cause of hypoxemia. We report the case of a patient who presented persistent hypoxemia after an adequate treatment for a severe asthma attack requiring intensive care unit admission. The patient underwent a transthoracic microbubbles contrast echocardiography that showed a massive patent foramen ovale. The favorable clinical course and the absence of major signs and symptoms related to patent foramen ovale allowed a conservative approach with a follow-up program. Patent foramen ovale should be suspected in case of persistent hypoxemia after a severe asthma attack had resolved.

**Electronic supplementary material:**

The online version of this article (10.1186/s13089-019-0121-z) contains supplementary material, which is available to authorized users.

## Background

Patent foramen ovale (PFO) is a common asymptomatic finding reflecting an embryonic remnant of fetal circulation with an estimated prevalence of 20–25% among adults [1].

Within a few minutes of giving birth, the increased pressure in left-side cameras and the lowering pulmonary pressure lead to foramen closure. However, if right-side heart pressure rapidly overcomes the left one, the foramen ovale may reopen, causing a right-to-left shunt [2].

Great attention has been paid to the role of PFO in the cryptogenic stroke, but also critically ill patients often present situations, such as ARDS, where it can be involved. Anyhow, it is not always easy to understand if PFO acts as incidental finding or represents an adaptive pathophysiological mechanism.

## Case presentation

A 20-year-old woman was admitted to the emergency department with a severe asthma attack. She had no relevant medical history except for minor asthma episodes successfully treated with short inhaled salbutamol cycles.

After treatment with bronchodilators and steroids, she was admitted to the intensive care unit (ICU) due to worsening clinical signs including a P_a_O_2_/F_I_O_2_ = 109 mmHg. During ICU stay, she underwent high flow nasal cannula ventilation with clinical improvement; however, SpO_2_ value while spontaneously breathing room air remained low for several days (P_a_O_2_/F_I_O_2_ < 200 mmHg). With the suspicion of pulmonary embolism, we performed a CT pulmonary angiography that did not show any pathological finding; therefore, we oriented our investigation to examine the presence of an intracardiac shunt.

**Fig. 1:**
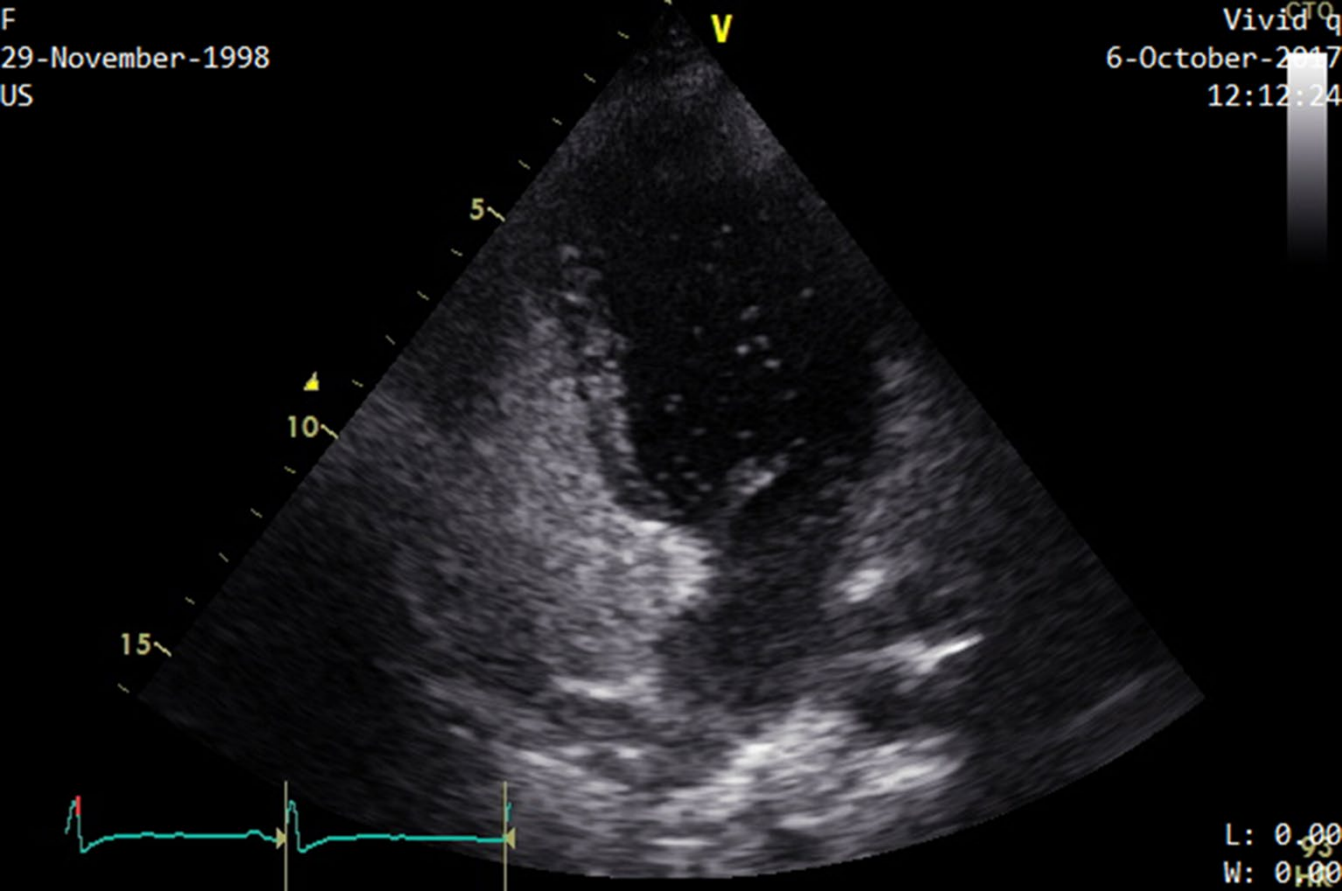
Transthoracic echocardiographic evaluation while the patient was normally breathing shows early microbubbles in left cardiac cameras

A transthoracic microbubbles contrast echocardiographic evaluation showed a PFO, with a massive shunt following Valsalva’s maneuver (Fig. 1. See also Additional file 1). Moreover, during TTE examination, no signs of acute right ventricular disfunction were highlighted, and estimating arterial pulmonary pressure was impossible due to the absence of tricuspidalic regurgitation. Normal left ventricular ejection fraction was observed.

According to a positive clinical evolution in the ICU and an improvement of the patient’s respiratory conditions, in agreement with cardiologists, we opted for a conservative approach and linked the patient into a follow-up program. After 6 months during follow-up, a control echocardiography was performed and it confirmed the persistence of PFO but with a significant reduction in its size.

Given the incidental PFO finding without major symptoms and the reduction in its size, cardiologists decided not to propose it for closure to prescribe neither anticoagulant nor antiplatelet therapy.

## Discussion

Patent foramen ovale is involved in many clinical situations, including cryptogenic stroke, decompression sickness, migraine, massive pulmonary embolism and acute cor pulmonale [3, 4]. Moreover, Boon et al. identified PFO as a risk factor for fatal outcome after severe asthma attack [5].

In the case here reported, the acute and transient increase in pulmonary pressure due to a severe asthma attack was probably responsible for the increased shunt through the foramen ovale, with low SpO_2_ values persisting for several days after the asthma attack was resolved.

Transthoracic echocardiographic evaluation (TTE) is the first approach in PFO diagnosis: transthoracic ultrasound both with color Doppler function and with microbubbles contrast has medium–high (46% to 78%) sensitivity, 99% of specificity in detecting PFO and its noninvasiveness and easiness to perform explains why it represents the first tool during diagnosis [6].

Transesophageal (TEE) evaluation increases sensitivity in PFO diagnosis up to 90% and represents the gold standard diagnostic technique; however, the acknowledged benefits should be weighed against the greater invasiveness and potentially complications [7, 8].

Transcranial Doppler is another diagnostic tool with high sensitivity and specificity of 97% and 93%, respectively, and could be used in addition to TEE examination to increase specificity and sensitivity while pre-operative studying before PFO closure [9].

Patent foramen ovale findings in the ICU could be asymptomatic or, as in the present case, the result of an adaptive response; however, PFO is a very frequent finding with uncertain significance in patients with severe ARDS and acute cor pulmonale [10].

It is also curiously reported that a right-to-left shunt can occur during normal right heart cameras’ pressures [11]. In the case here described, we did not find indirect signs of pulmonary hypertension such as abnormal TAPSE or right ventricular enlargement; however, we reasoned that PFO could have lowered right atrial pressure as well as a good left ventricular ejection fraction kept end diastolic pressure low, avoiding secondary pulmonary hypertension due to high left atrial pressure.

The question “What should I do if PFO is diagnosed?” often implies PFO closure in young patients after cryptogenic stroke. However, the debate about what to do when PFO occurs as an incidental finding is ongoing.

Ng et al. described the increased risk of peri-operative cerebral ischemic stroke in patients for whom PFO was diagnosed during a pre-operative examination [10].

Treatment options include medical therapy—mainly antiplatelet therapy or anticoagulants—and percutaneous PFO closure or via cardiac surgery if the atrium has to be opened as part of a scheduled surgical procedure. There remains a lack of strong evidence that the incidence of stroke is significantly reduced after PFO closure, although recent trials have shown several advantages to the procedure [12].

## Conclusions

In conclusion, the presence of PFO should be highly suspected and looked for as potential cause of unexpected persistent hypoxemia after severe acute asthma attack; echographic evaluation represents an important bedside tool during diagnostics of critically ill patients with unexplained persistent hypoxemia.

A follow-up program is suggested to establish if a conservative or interventional treatment is needed when a PFO is diagnosed as incidental finding.

## Additional file

**Additional file 1.** Transthoracic echocardiographic videoclip while the patient performs Valsalva’s manoeuvre shows a massive shunt.

## Abbreviations

PFO: patent foramen ovale
ICU: intensive care unit
ARDS: acute respiratory distress syndrome
TTE: transthoracic echocardiographic evaluation
TEE: transesophageal echocardiographic evaluation
TCD: transcranial Doppler
